# Characteristics of depression, anxiety, impulsivity, and aggression among various types of drug users and factors for developing severe depression: a cross-sectional study

**DOI:** 10.1186/s12888-022-03933-z

**Published:** 2022-04-19

**Authors:** Dan Luo, Lu Tan, Danlin Shen, Zhe Gao, LiangMing Yu, Mingfeng Lai, Jiajun Xu, Jing Li

**Affiliations:** 1 Mental Health Center, West China Hospital of Sichuan University, 28 Dian Xin Nan Jie, Chengdu, 610041 Sichuan China; 2 Sleep Medicine Center, West China Hospital of Sichuan University, Chengdu, China

**Keywords:** Methamphetamine, Heroin, Severe depression, Impulsivity, Aggression

## Abstract

**Background:**

Mood disorder, impulsivity and aggression are common in drug users compared to healthy controls. However, no study has focused on the difference in various types of drug users. Therefore, the objective of this study was to explore the differences in depression, anxiety, impulsivity, and aggression among methamphetamine, heroin and polysubstance users and to further explore the risk factors for severe depression in the three groups.

**Methods:**

Drug users over 18 years old who met the DSM-V diagnostic criteria for substance -related disorders were included in the study. All participants completed a general questionnaire, the Zung Self-Rating Depression Scale (SDS), the Zung Self-Rating Anxiety Scale (SAS), Barratt impulsiveness Scale Version 11 (BIS-11), and the Buss-Perry Aggression Questionnaire (BPAQ). One-way ANOVAs or Chi-square tests were used to test the differences among the groups, correlation analysis was used to test the relationship between drug use and other parameters, and multiple logistic regression was conducted to assess the risk factors for severe depression.

**Results:**

A total of 1,486 participants were included, comprising 86.3% males with a mean age of 38.97 years. There was a significant difference in the percentage of severe depression and SDS scores among the three groups, but no significant difference was found in SAS, BIS-11 and BPAQ scores. Using methamphetamines, hostility and anxiety were risk factors for developing severe depression in all the participants and anxiety remained constant in the other three groups. Moreover, methamphetamine use was 2.16 and 3.35 times more likely to cause severe depression than heroin and polysubstance use, respectively. The initial age of substance use was negatively correlated with BPAQ, SAS, and SDS scores, whereas the drug use duration and addiction duration were positively correlated.

**Conclusions:**

In this study, we found that the highest prevalence of severe depression was in participants using methamphetamines and that using methamphetamines, hostility, and anxiety were risk factors for developing severe depression. This result addressed an important gap in our knowledge of the different characteristics of depression, anxiety, impulsivity and aggression in various types of substance users and provides clinicians and policy-makers with directions for intervention and preventing relapse.

## Background

Drug abuse issues have become a social problem that affects millions of people with the development of the economy. According to the World Drug Report 2019, nearly 270 million people used drugs, 35 million were addicted to drugs, and 600,000 died from drug abuse that year around the world [1]. The situation was also tough in China with a total of 2.148 million drug users registered. Among them, 55.2% were methamphetamine users, 37.5% were heroin users, and 2.3% were ketamine users at the end of 2019 [2].

Mental disorders, especially mood disorders, are common in drug abuse patients. Previous studies have found a bidirectional relationship between mood disorders such as depression or anxiety and drug abuse [3–5]. Grant et al. reported that 20% of individuals who have substance use disorders presented with at least one independent mood disorder in the US [6]. Similarly, another study found that 24% to 43% of patients with anxiety disorders have a lifetime history of substance use [7]. Regarding mood disorders in different drug addiction groups, an early study reported that Beck’s Depression Inventory score was significantly higher in the heroin addiction group than in the recreational heroin use group, but there was no difference on Beck’s Anxiety Inventory score [8]. Another study also found that the use of marihuana increased the risk of anxiety, depression and suicide tendencies among juveniles [9].

Impulsive behaviours such as impulsivity and aggression were also common in drug abuse patients. Impulsivity could promote the initiation, maintenance and relapse of drug addiction, which typifies the at-drug abuser group [10–12]. Compared to age- and sex-matched healthy controls, a higher level of impulsivity was found in methamphetamine users with brief abstinence (no use for 2 days) [13]. A higher Barratt impulsiveness scale version 11 (BIS-11) score was also found in methamphetamine users seeking treatment than in cocaine users, who scored consistently higher than healthy controls [14]. Moreover, another study found deficits in reflection impulsivity [15], response inhibition, and delay discounting [16] but no differences in motor and nonplanning impulsivity in heroin users [17]. Regarding the relationship between substance addiction and aggression, previous studies suggested that marijuana [18], heroin [19], and methamphetamine abusers [20] perform more aggressively and may directly increase the occurrence of aggressive behaviour. Drug abuse may induce various forms of aggressive behaviour during drug intoxication [21, 22], and the severity of aggressive behaviour is time-dependent [23].

A relationship between drug use, mood disorders and impulsive behaviour was also found. Studies have suggested that individuals who use drug such as marijuana [18], heroin [19], and methamphetamines [20] have higher aggression and impulsivity scores. Zorick et al. also confirmed that anxiety and depression were the most important psychological factors for withdrawal symptoms and cravings among drug users [24] and patients with comorbidity of mood disorder and drug use had a higher risk of suicide [25]. Furthermore, Coryell et al. found that recent aggressive behaviour and higher levels of impulsivity were risk factors for suicide in a group of patients suffering from major depressive disorder [26].

To our knowledge, the relationship among drug use, mood disorders and impulsive behaviour has not been studied before. We speculated that drug abuse may induce mood disorder such as depressive, anxiety symptoms and emotion regulation difficulties, which may in turn cause impulsive behaviours, such as gambling and using alcohol and drugs. Moreover, previous studies only compared the prevalence of mood disorders and impulsive behaviour in a specific drug user (such as methamphetamines, heroin, or marijuana) compared to healthy controls. The difference in mood disorder and impulsive behaviour among various types of drug users, especially among a group with compulsory detoxification has not been studied. This research may provide clues for individualized therapy, preventative strategies, and specific management measures in different drug users. Therefore, the purpose of the study was to explore differences and the relationships among depression, anxiety, impulsivity and aggression and to further explore the risk factors for the development of severe depression in various types of drug users.

## Methods

### Study design and setting

This cross-sectional study examined depression, anxiety, impulsivity, and aggression among methamphetamine-only, heroin-only, and polysubstance groups. All clinical data were collected from August 2016 to July 2018 at the Compulsory Detoxification Centers in Sichuan, Shaanxi, Qinghai, Gansu, Ningxia Province. A face-to-face interview was conducted by experienced psychiatrists. This study was approved by the West China Hospital of Sichuan University Biomedical Research Ethics Committee. All the participants who were willing to participate in this study were informed of the study purpose, methods, and possible risks and benefits they could receive from this study in advance. Those who agreed to participate signed the informed consent form.

### Participants

**Fig. 1:**
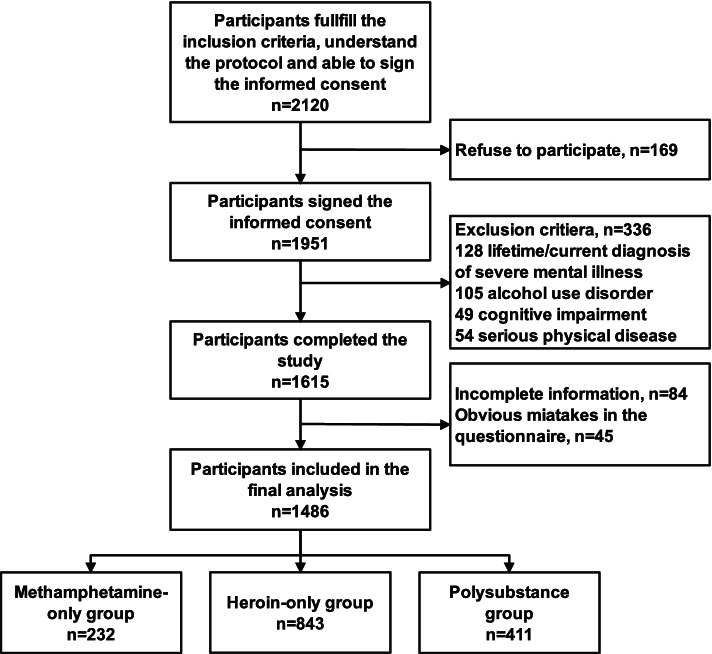
Flowchart of the study

Participants who were over 18 years old and diagnosed with a substance-related disorder according to DSM-V criteria with recent usage of methamphetamine or heroin and detoxified for at least two weeks at the time of enrolment were invited for screening. They should also understand the study protocol and be able to sign the informed consent form. Participants with lifetime/current diagnosis of severe mental illness (SMI) according to the Standards for the Management and Treatment of Severe Mental Disorders in China (including schizophrenia, bipolar disorder, paranoid psychosis, intellectual disability, epileptic mental disorder, and schizoaffective disorder) or personality disorder, alcohol use disorder, cognitive impairment, and any other serious physical diseases were excluded. A participant flow chart is presented in Fig. 1. Participants who used heroin only were defined as the heroin-only group, and those who use methamphetamine only were defined as the methamphetamine-only group. The polysubstance group was defined as participants who used more than one drug ever, and participants who were divided into the polysubstance group mainly used both heroin and methamphetamine in our study.

### Measurements

#### General questionnaire

Age, gender, ethnicity, education, employment status, type of substance use, initial age of substance use, substance use duration and substance addiction duration were collected from all participants. Participants who reported that they had repetitive and compulsive self-medication behaviour with increasing use of substance doses were considered as addicted. The substance addiction duration was the time they had the above behaviours to the time when they participated in this study. Addiction duration for those who did not have the above behaviours was 0 years.

#### Zung Self-Rating Depression Scale and Zung Self-Rating Anxiety Scale

The Zung Self-Rating Depression Scale (SDS) and Zung Self-Rating Anxiety Scale (SAS) were used to assess the presence and severity of depression and anxiety in all participants [27, 28]. The SDS and SAS are widely used in substance users. The questionnaire comprises 20 depression- or anxiety-related items rated from 1 to 4. The total score was calculated by the sum of each item, and the standardized total score was the total score multiplied by 1.25 (ranging from 25 to 100). The Chinese criteria for mild, moderate and severe depression were 53–62, 63–72, and > 72, respectively. The criteria for mild, moderate, and severe anxiety were 50–60, 61–70, and > 70, respectively.

#### Barratt Impulsiveness Scale version 11

Impulsivity was measured by the Chinese version of Barratt Impulsiveness Scale version 11 (BIS-11) [29, 30], which is the most widely used questionnaire to assess impulsiveness. The BIS-11 contains 30 items that assess three aspects of impulsivity, including attentional impulsivity (AI, i.e., defect in thought or difficulty in completing task), motor impulsivity (MI, i.e., take action without consideration) and nonplanning impulsivity (NPI, i.e., lack of future planning and tendency to live an irregular lifestyle). Each item ranges from 1 (never) to 5 (always). The range of impulsivity is defined as high impulsivity (> = 72), normal (52–71) and low impulsivity (< = 51) according to a previous study [31].

#### Buss-Perry Aggression Questionnaire

The Buss-Perry Aggression Questionnaire (BPAQ) is a self-reported scale to evaluate four domains of aggression, including physical aggression, verbal aggression, anger and hostility. The Chinese version of the BPAQ we used in the study was revised by Lv et al. [32], and is made up of 22 items. Each question is answered on a Likert scale of 1 (extremely uncharacteristic of me) to 5 (extremely characteristic of me). Higher scores indicate more severe degrees of aggressive temperament.

### Statistical analysis

Continuous variables are presented as the mean ± standard deviation, while categorical variables are presented as the number (N) and percentage (%). Comparisons of continuous variables and proportions among groups were performed using one-way ANOVA followed by the LSD post-hoc test and chi-square test. The homoscedasticity of the data herein was tested prior to one-way ANOVA. Pearson correlation analysis was used to evaluate the correlation between substance use vs. depression, anxiety, impulsivity, and aggression. Univariate logistic regression analysis was performed before multivariate logistic regression analysis to determine the possible risk factors related to severe depression. Independent variables with *p* < 0.2 in the univariable analysis were included in the multivariable analysis. Statistical analyses were performed using SPSS software (version 26.0), and *p* < 0.05 was defined as statistically significant.

## Results

### Demographic characteristics

A total of 2120 participants were invited to participate in the study and 1486 were available for the final analysis, including 232 participants in the methamphetamine-only group, 843 participants in the heroin-only group and 411 participants in the polysubstance group. A participant flow chart is illustrated in Fig. 1. The demographic characteristics of the three groups are presented in Table 1. The majority of the participants were male (86.3%), and the distribution was also similar in the three separate groups. The mean age and initial age of the heroin-only group were significantly older than those of the other two groups. However, substance use and addiction duration showed no significant difference (*p* = 0.18 and *p* = 0.82, respectively).

**Table 1:** Demographic characteristics in the methamphetamine-only, heroin-only, and polysubstance groups

	Methamphetamine-only	Heroin-only	Poly-substance	*P* value
	(*N* = 232)	(*N* = 843)	(*N* = 411)	
Provinces				** < 0.001**
Sichuan	13 (5.6%)	66 (7.8%)	120 (29.2%)	
Shaanxi	39 (16.8%)	206 (24.4%)	94 (22.9%)	
Qinghai	101 (43.5%)	167 (19.8%)	91 (22.1%)	
Gansu	23 (9.9%)	254 (30.1%)	51 (12.4%)	
Ningxia	56 (24.1%)	150 (17.8%)	55 (13.4%)	
Gender				**0.002**
Male	189 (81.5%)	750 (89.0%)	344 (83.7%)	
Female	43 (18.5%)	93 (11.0%)	67 (16.3%)	
Age, years	31.26 ± 8.02	43.46 ± 7.65	34.13 ± 9.53	** < 0.001**
Ethnicity				**0.005**
Han	168 (72.4%)	649 (77.0%)	341 (83.0%)	
Minority	64 (27.6%)	194 (23.0%)	70 (17.0%)	
Full-time education				**0.03**
≤ 9 years	184 (79.2%)	632 (75.0%)	288 (70.1%)	
> 9 years	48 (20.8%)	211 (25.0%)	123 (29.9%)	
Employment status				** < 0.001**
Not employed	113 (48.7%)	540 (64.1%)	232 (56.4%)	
Employed	119 (51.3%)	303 (35.9%)	179 (43.6%)	
Initial age of substance use, years	23.98 ± 8.06	35.44 ± 9.63	26.86 ± 9.91	** < 0.001**
Substance use duration, years	7.28 ± 5.85	8.01 ± 6.34	7.27 ± 5.82	0.18
Substance addiction duration, years	4.35 ± 4.50	4.99 ± 5.54	4.73 ± 5.08	0.82

Continuous data are presented as the mean ± standard deviation, and categorized data are presented as numbers (percentage)

### Depression and anxiety in different types of drug users

**Fig. 2:**
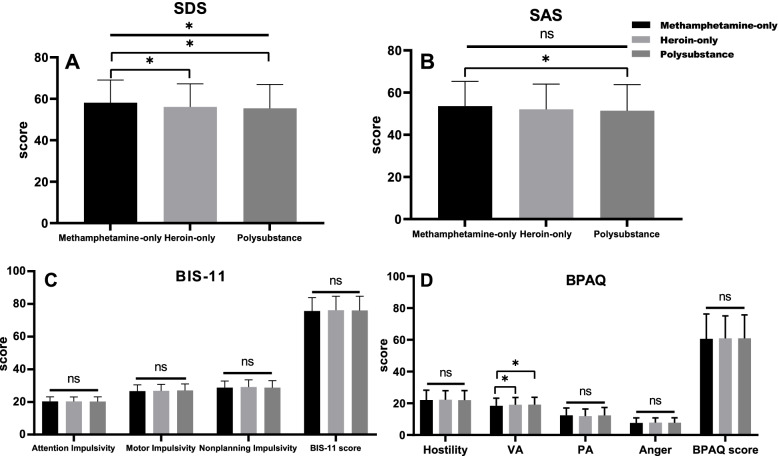
Differences of in BIS-11, BPAQ, SDS, and SAS scores among the methamphetamine-only, heroin-only, and polysubstance groups. *BIS-11* Barratt impulsiveness scale version 11, *VA* verbal aggression, *PA* physical aggression, *BPAQ* Buss-Perry aggression questionnaire, *SDS* Zung Self-Rating Depression Scale, *SAS* Zung Self-Rating Anxiety Scale, *ns* no significance. *p < 0.05 by one-way ANOVA

As shown in Table 2, more than two-thirds of participants presented with depression and anxiety in the three groups. The percentage of severe depression evaluated by SDS was the highest in the methamphetamine-only group, middle in the heroin-only group, and the lowest in the polysubstance group (9.5% vs. 5.6% vs. 3.9%, *p* = 0.005). The mean SDS score of the methamphetamine-only group was 58.06 ± 10.94, which was higher than that of the heroin-only group (56.06 ± 11.12, *p* = 0.02) and the polysubstance group (55.36 ± 11.58, *p* = 0.004), and there was no difference between the heroin-only group and the polysubstance group (*p* > 0.05) (Fig. 2A).

**Table 2:** The percentage of different severities of depression and anxiety among the three groups of substance users

	Methamphetamine-only	Heroin-only	Poly-substance	*P* value
	(*N* = 232)	(*N* = 843)	(*N* = 411)	
Anxiety				0.07
No	84(36.2%)	355(42.1%)	188(45.7%)	
Mild	67(28.9%)	262(31.1%)	115(28.0%)	
Moderate	63(27.2%)	157(18.6%)	77(18.7%)	
Severe	18(7.8%)	69(8.2%)	31(7.5%)	
Depression				**0.005**
No	54(23.3%)	262(31.1%)	141(34.3%)	
Mild	91(39.2%)	309(36.7%)	129(31.4%)	
Moderate	65(28.0%)	225(26.7%)	125(30.4%)	
Severe	22(9.5%)	47(5.6%)	16(3.9%)	

Depression and anxiety were measured by the Zung Self-Rating Depression Scale and the Zung Self-Rating Anxiety Scale. The chi-square test was used for comparisons among groups

For anxiety, there was no significant difference in the percentage of severe anxiety evaluated by SAS in the three groups (7.8% vs. 8.2% vs. 7.5%, *p* = 0.07). In Fig. 2B, the mean SAS score of the methamphetamine-only group was significantly higher than that of the polysubstance group (53.57 ± 11.76 vs. 51.39 ± 12.38, *p* = 0.03). However, no significant difference was found between the heroin-only group (52.09 ± 11.92) and the methamphetamine-only group or the polysubstance group.

### Impulsivity and aggression in different types of drug users

The BIS-11 score is shown in Fig. 2C. The total mean scores of the methamphetamine-only group, heroin-only group, and polysubstance group were 75.65 ± 8.17, 76.13 ± 8.59, and 75.00 ± 8.71, respectively, which showed no significant difference (*p* = 0.75). In addition, the scores on attention (*p* = 0.97), motor (*p* = 0.44) and nonplanning impulsivity (*p* = 0.29) showed no significant differences among the three groups.

As shown in Fig. 2D, the mean total BPAQ score showed no significant differences among the three groups (60.56 ± 15.71 vs. 60.87 ± 14.18 vs. 61.24 ± 15.30, *p* = 0.84). In addition, the hostility, verbal aggression, physical aggression, and anger domains also showed no significant differences among the three groups. However, the verbal aggression score was significantly lower in the methamphetamine-only group than in the heroin-only group (18.4 ± 4.79 vs. 19.08 ± 4.51, *p* = 0.05) and the polysubstance group (18.4 ± 4.79 vs. 19.17 ± 4.60, *p* = 0.04).

### Correlation between substance use, mood status, impulsivity, and aggression

**Fig. 3:**
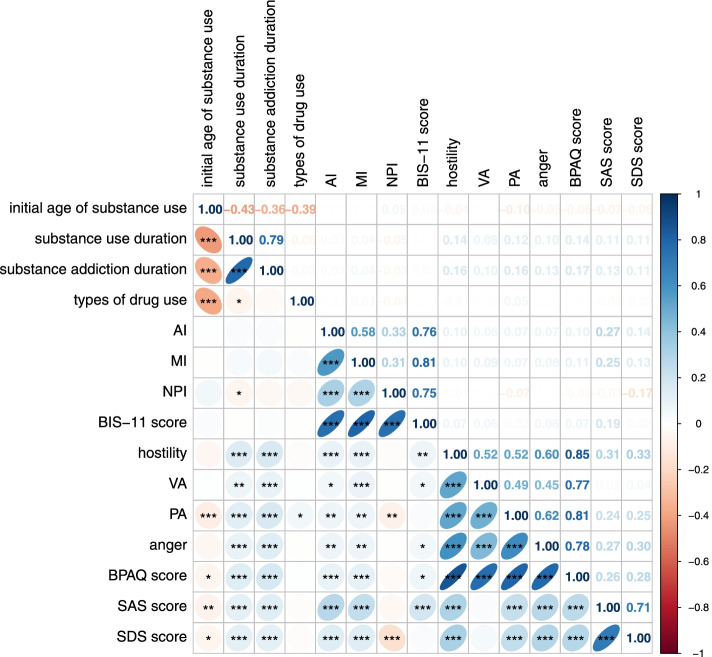
Correlations between substance use and impulsivity, aggression, anxiety and depression. *VA* verbal aggression, *PA* physical aggression, *BPAQ* Buss-Perry aggression questionnaire, *AI* attention impulsivity, *MI* motor impulsivity, *NPI* nonplanning impulsivity, *BIS-11* Barratt impulsiveness scale version 11, *SDS* Zung Self-Rating Depression Scale, *SAS* Zung Self-Rating Anxiety Scale. *p < 0.05, **p < 0.01, ***p < 0.001

As shown in Fig. 3, nonplanning impulsivity presented a slightly negative correlation (coefficient of 0.05, 95% CI -0.10 to 0.006) with substance use duration. The SDS and SAS scores were negatively correlated with the initial age of the substance and positively correlated with substance use duration and substance addiction duration. In addition, the total BPAQ score and score on each domain had a positive correlation with substance use duration and addiction duration, whereas only the total BPAQ score and physical aggression score were negatively correlated with the initial age of substance use. The SDS and SAS scores were slightly to moderately correlated with the BIS-11 score and BPAQ score (coefficients ranged from 0.19 (95% CI 0.14 to 0.24) to 0.28 (95% CI 0.23 to 0.33)).

### Risk factors for severe depression in drug users

In Table 3, we revealed that using methamphetamine, the hostility score and the SAS score were risk factors, whereas the nonplanning impulsivity score was a protective factor for severe depression in all participants. In addition, the odds of developing severe depression were 2.16 times and 3.35 times higher in participants using methamphetamine than in participants using heroin and polysubstance. The relative risk of severe depression increased by 0.08 and 0.16 for every one-unit increase in the hostility score and SAS score, respectively. For the three separate groups (Table 4), the SAS score remained constant as a risk factor for severe depression. The hostility score and anger score from the BPAQ were also risk factors for severe depression in the heroin-only group and polysubstance group, respectively. Moreover, the nonplanning impulsivity score was a protective factor for severe depression in the polysubstance group.

**Table 3:** Risk factors for severe depression in all participants

Independent variables	All participants			
	Univariable analysis		Multivariable analysis	
	OR (95%CI)	*P*	OR (95%CI)	*P*
Sex				
Male vs. female	0.72 (0.41, 1.29)	0.27		
Ethnicity				
Minority vs. Han	0.75 (0.42, 1.32)	0.31		
Full-time education				
≤ 9 years vs. > 9 years	0.82 (0.51, 1.33)	0.42		
Employment status				
Not employed vs. employed	1.13 (0.72, 1.78)	0.59		
Types of drug use				
Methamphetamine vs. Heroin	1.77 (1.05, 3.01)	0.03	2.16 (1.16, 4.02)	**0.02**
Methamphetamine vs. Polysubstance	2.59 (1.33, 5.03)	0.005	3.35 (1.55, 7.25)	**0.002**
Initial age of substance use	1.00 (0.98, 1.02)	1.00		
Substance use duration	1.02 (0.99, 1.05)	0.28		
Substance addiction duration	1.03 (0.99, 1.07)	0.12	0.99 (0.95, 1.04)	0.70
BIS-11 score				
Attention impulsivity score	1.16 (1.07, 1.26)	< 0.001	0.99 (0.89, 1.10)	0.85
Motor impulsivity score	1.10 (1.05, 1.17)	< 0.001	1.04 (0.96, 1.13)	0.32
Nonplanning impulsivity score	0.95 (0.90, 1.00)	0.03	0.90 (0.84, 0.95)	**0.001**
BPAQ score				
Hostility score	1.14 (1.09, 1.18)	< 0.001	1.08 (1.01, 1.16)	**0.02**
Verbal aggression score	1.04 (0.99, 1.10)	0.09	0.99 (0.91, 1.07)	0.72
Physical aggression score	1.08 (1.03, 1.13)	0.001	0.96 (0.90, 1.04)	0.30
Anger score	1.19 (1.10, 1.28)	< 0.001	1.06 (0.94, 1.20)	0.31
SAS score	1.16 (1.13, 1.19)	< 0.001	1.16 (1.12, 1.19)	** < 0.001**

Independent variables with *p* < 0.2 in the univariable analysis were included in the multivariable model

*BIS-11* Barratt impulsiveness scale version 11, *BPAQ* Buss-Perry aggression questionnaire, *SAS* Zung Self-Rating Anxiety Scale. Severe depression was defined as the Zung Self-Rating Depression Scale score > 72

**Table 4:** Risk factors for severe depression in different drug user groups

Independent variables	Methamphetamine-only				Heroin-only				Polysubstance			
	Univariable analysis		Multivariable model		Univariable analysis		Multivariable model		Univariable analysis		Multivariable model	
	OR (95%CI)	*P*	OR (95%CI)	*P*	OR (95%CI)	*P*	OR (95%CI)	*P*	OR (95%CI)	*P*	OR (95%CI)	*P*
Sex												
Male vs. female	1.49(0.42, 5.28)	0.54			0.69(0.30, 1.59)	0.39			0.41(0.14, 1.22)	0.11	0.12(0.02, 0.62)	**0.01**
Ethnicity												
Minority vs. Han	0.75(0.27, 2.13)	0.59			0.57(0.25, 1.29)	0.18	0.38(0.15, 0.97)	**0.04**	1.13(0.31, 4.07)	0.85		
Full-time education												
≤ 9 years vs. > 9 years	1.19(0.38, 3.70)	0.76			0.70(0.37, 1.31)	0.26			0.70(0.25, 1.97)	0.50		
Employment status												
Not employed vs. employed	1.59(0.65, 3.88)	0.31			1.21(0.64, 2.27)	0.55			0.76(0.28, 2.08)	0.60		
Initial age of substance use	0.98(0.93, 1.04)	0.51			1.01(0.98, 1.04)	0.56			1.02(0.97, 1.07)	0.57		
Substance use duration	1.05(0.99, 1.13)	0.13	1.03(0.91, 1.18)	0.62	1.00(0.96, 1.05)	0.88			1.02(0.95, 1.11)	0.55		
Substance addiction duration	1.07(0.99, 1.16)	0.10	1.01(0.85, 1.19)	0.95	1.02(0.97, 1.07)	0.53			1.05(0.96, 1.14)	0.29		
BIS-11 score												
Attention impulsivity score	1.13(0.96, 1.32)	0.15	0.93(0.74, 1.16)	0.51	1.20(1.08, 1.33)	0.001	1.01(0.88, 1.17)	0.89	1.13(0.95, 1.34)	0.18	1.02(0.77, 1.36)	0.88
Motor impulsivity score	1.12(1.00, 1.26)	0.05	1.06(0.90, 1.25)	0.47	1.09(1.01, 1.17)	0.03	0.99(0.89, 1.09)	0.77	1.16(1.03, 1.32)	0.02	1.23(0.97, 1.56)	0.09
Nonplanning impulsivity score	0.99(0.89, 1.10)	0.86			0.94(0.88, 1.01)	0.07	0.93(0.85, 1.00)	0.06	0.92(0.82, 1.03)	0.13	0.70(0.56, 0.87)	**0.001**
BPAQ score												
Hostility score	1.08(1.00, 1.17)	0.04	1.01(0.88, 1.15)	0.91	1.16(1.01, 1.23)	< 0.001	1.09(1.01, 1.19)	**0.03**	1.15(1.06, 1.26)	0.002	1.04(0.89, 1.20)	0.64
Verbal aggression score	1.09(0.99, 1.20)	0.09	1.11(0.94, 1.31)	0.23	1.03(0.96, 1.10)	0.38			1.04(0.93, 1.17)	0.46		
Physical aggression score	1.04(0.95, 1.15)	0.37			1.10(1.04, 1.17)	0.003	0.98(0.89, 1.08)	0.66	1.08(0.98, 1.19)	0.11	0.89(0.77, 1.04)	0.15
Anger score	1.09(0.95, 1.26)	0.23			1.19(1.08, 1.31)	0.001	0.95(0.81, 1.11)	0.51	1.33(1.13, 1.58)	0.001	1.55(1.10, 2.19)	**0.01**
SAS score	1.16(1.10, 1.23)	< 0.001	1.17(1.10, 1.25)	** < 0.001**	1.17(1.12, 1.21)	< 0.001	1.17(1.12, 1.22)	** < 0.001**	1.15(1.09, 1.20)	< 0.001	1.21 (1.12, 1.30)	** < 0.001**

Independent variables with p < 0.2 in the univariable analysis were included in the multivariable model

BIS-11, Barratt impulsiveness scale version 11; BPAQ, Buss-Perry aggression questionnaire; SAS, Zung Self-Rating Anxiety Scale. Severe depression was defined as the Zung Self-Rating Depression Scale score > 72

## Discussion

In this cross-sectional study, we explored the differences in depression, anxiety, impulsivity and aggression and risk factors for severe depression in methamphetamine-only, heroin-only, and polysubstance groups among 1486 participants. We found that the prevalence of severe depression (defined as an SDS score > 72) was the highest in the methamphetamine-only group, middle in the heroin-only group, and lowest in the polysubstance group (9.5% > 5.6% > 3.9%, *p* = 0.005). However, most components of impulsivity and aggression scores showed no significant difference among the three groups. Drug use types were a risk factor for severe depression in all participants, as they were 2.16 times and 3.35 times more likely to develop severe depression in the methamphetamine-only group than in the heroin-only and polysubstance groups, respectively. Moreover, the SDS and SAS scores were correlated with the initial age of substance use, substance use duration, substance addiction duration, impulsiveness and aggression evaluated by the BIS-11 and BPAQ respectively. The aggression evaluated by the BPAQ was also correlated with substance addiction duration.

The high prevalence of depressive symptoms among substance users in this study was in line with a previous study that found that 57.6% of participants presented with any type of psychiatric symptoms including depressive, anxiety and psychosis symptoms among 1277 methamphetamine users [33]. Another study conducted by Le et al. also found that 21% of heroin abusers reported having major depressive disorder [34]. However, the prevalence of severe depression in the heroin-only group was much lower, at 5.6%, in our study. One possible reason may be related to the longer withdrawal time in our study compared to others, which may reduce the effect of the drug on mood. Another reason may be based on the different evaluation methods. Some studies used a self-report questionnaire (SDS), and some used a questionnaire evaluated by experienced doctors (Beck’s Depression Scale or Hamilton Depression Scale). The different criteria for depression may also have an impact on prevalence because depression was diagnosed by experienced psychiatrists in some studies and was defined by the SDS score in our study.

In our study, we found that participants with methamphetamine abuse had a significantly higher risk of developing severe depression than those with heroin abuse or polysubstance abuse. One recent meta-analysis also indicated that methamphetamine use was associated with a 1.3-fold increased risk of developing depression compared with no methamphetamine use after controlling for demographic characteristics, other substance use, and premorbid risk [35]. A study conducted by Le et al. revealed that heroin use was less likely to result in major depressive disorder, which was partly consistent with our study [34]. Heroin and methamphetamine are two different addictive drugs. Methamphetamine can directly damage dopamine neurons [36], resulting in withdrawal, whereas heroin mainly converts to morphine and binds to μ-opioid receptors, resulting in analgesic and anxiolytic effects [37]. One of the possible pathophysiological mechanisms for the higher prevalence of severe depression with methamphetamine use is the disruption of circadian rhythms. A study [38] reported that the prokineticin 2 receptor gene (PROKR2), which has been shown to be essential for circadian rhythm [39], is a common susceptibility gene for methamphetamine dependence and mood disorders. In addition, acute methamphetamine use activates brain reward system and results in feelings of pleasure and euphoria [40]. However, repetitive use of methamphetamine leads to neurotoxic effects, such as dysregulation of neurotransmitters, and neurite degeneration in the reward system [41]. A study using positron emission tomography has shown that the use of methamphetamine may lead to a sustained decrease in the density of brain dopamine transporters, which may relate to the long persisting anhedonia and other depressive symptoms after the last use [42]. Moreover, other neuroimaging studies have confirmed brain function changes in the reward system, especially in the striatum and limbic and paralimbic regions, which contribute to depressive symptoms [43, 44]. In addition to types of drug use, anxiety evaluated by the SAS was also a risk factor for developing severe depression in all the participants and in the three groups. This finding was consistent with what was found by Zhang et al., suggesting that anxiety was the first predictor of depressive symptoms in methamphetamine users [45]. Moreover, the hostility score was also a risk factor for developing severe depression in all participants and in the heroin-only group. A previous study found that the severity of depression was positively associated with BPAQ scores and hostility scores in adolescents [46].

The prevalence of different severities of anxiety was not significantly different among the three groups, and the SAS score was higher in the methamphetamine-only group than in the polysubstance group. This result is inconsistent with a previous study that reported that methamphetamine abusers have higher anxiety (evaluated by the Hamilton Anxiety Rating Scale) than heroin abusers [47]. The interpretation of this difference may be based on different assessment tools. Although the impulsivity evaluated by the BIS-11 and aggression evaluated by the BPAQ showed no significant differences among the three groups, the scores were also higher than those of normal people. A cross-sectional study also found that both heroin and methamphetamine abuse were more aggressive than normal controls [48]. The possible reason for the lack of difference in methamphetamine users and heroin users is that participants experienced long abstinence duration. In this study, we found a negative correlation between initial age of drug use and depression or anxiety. However, there were no consistent results in previous studies. Some studies suggested that the initial age of drug use was a protective factor for mental symptoms [49], whereas others found no association [45]. Moreover, substance addiction duration was positively correlated with mood disorders and aggression, which was in line with previous studies. A dose–response relationship between methamphetamine use duration and the risk of depressive symptoms was found, with odds ratios of 1.74 times higher in 1- to 5-year methamphetamine users and 2.07 times higher in ≥ 5-year methamphetamine users than in < 1-year methamphetamine users [50]. Another study including 1,580 arrestees in California found that methamphetamine-dependent patients were more likely to report depressive symptoms and suicidal ideation than those who denied methamphetamine use [51]. A correlation between mood disorder and impulsivity or aggression was also found. Zhang et al. revealed that depression is positively correlated with total BIS-11, attention impulsivity, motor impulsivity and nonplanning impulsivity scores [45]. However, Swann et al. suggested that motor impulsivity, is associated with mania in patients with bipolar disorder but not with depression [52]. All these findings implicated a bidirectional correlation among substance use, mood disorders, impulsivity, and aggression.

Studies have indicated that individuals who are young men and have a low educational level, low income, and unemployment status are more likely to take drugs [53], and these characteristics are also strongly associated with impulsive and aggressive behavior [54]. In this study, the participants were mainly male (86.3%), with middle or low educational attainment (74.2%), and unemployed (69.6%). The age of 70.5% of the participants ranged from 16 to 45 years old. We also found that the age and the initial age of substance use of the methamphetamine-only group was younger than that of the heroin-only group, which was consistent with the current situation of substance use in China. In terms of seized drugs, methamphetamine and heroin are currently the main circulating drugs in China. In addition, studies have found that humans who use methamphetamine often have problems with poly substance abuse as a way to mitigate the side effects of methamphetamines, including stimulants (such as cocaine) [55], tranquilizers, and opioids [56].

These findings have implications for clinicians and policy-makers. For clinicians, the meaning of these findings is that they need to assess not only levels of severity of substance use disorders but also mood disorders that may be a cause for relapse when treating patients with substance use disorders. For policy-makers, more mental health services should be offered to patients with substance use disorder, and targeted strategies for various types of drug users should be conducted to prevent relapse.

There are some limitations that may affect the interpretation of the results. First, due to the different types of drugs and various dosages, it was difficult to estimate the quantities of drug-taking. Therefore, the effect of drug use frequency and dosage on mood and behaviour disorders is unknown. Second, because of some social problems, substance abusers may be more likely to conceal some information, which may affect the accuracy of the data. Third, the samples were from compulsory detoxification centres, which that may limit the generalizability of conclusions to other populations. Fourth, the estimates reported in our article were based on SDS and SAS cut-offs rather than clinical appraisal, and they should be interpreted with caution as depression and anxiety prevalence estimates in drug users. Finally, the group of methamphetamine users was considerably smaller than the heroin and polysubstance use groups, which could have affected the statistical power to detect relevant determinants of severe depression in the regression analyses presented in our study.

## Conclusions

The current study was the first to compare the differences in characteristics, mood disorders, impulsivity, and aggression in various types of drug users and to explore the risk factors for the development of severe depression. We found a higher prevalence of severe depression in participants using methamphetamine than in those using heroin or using multiple substances, and methamphetamine abuse, anxiety, and hostility were risk factors for developing severe depression in substance users. Anxiety remained a steady risk factor for developing severe depression in drug users. These results addressed an important gap in our knowledge of the different characteristics of depression, anxiety, impulsivity, and aggression among various types of substance use and provide a clue for further study.

## Data Availability

The data used during the current study are available from the corresponding author on reasonable request.

## Abbreviations

AI: Attentional impulsivity
BIS-11: Barratt impulsiveness scale version 11
BPAQ: Buss-Perry aggression questionnaire
METH: Methamphetamine
MI: Motor impulsivity
NPI: Nonplanning impulsivity
PA: Physical aggression
SAS: Zung Self-Rating Anxiety Scale
SDS: Zung Self-Rating Depression Scale
VA: Verbal aggression
